# Building improvement capacity in mental health services

**DOI:** 10.1192/bji.2020.27

**Published:** 2020-11

**Authors:** Charles Vincent

**Affiliations:** 1 Director, Oxford Healthcare Improvement, Oxford Health NHS Foundation Trust, UK. Email: charles.vincent@psy.ox.ac.uk

**Keywords:** Patient safety, quality improvement, mental health services, education and training, community mental health teams

## Abstract

Improving the delivery of existing treatment may often bring much greater benefits than developing new treatments and technologies. To achieve this, clinical teams and organisations need to build capacity for sustained and systematic improvement. Organisations can build improvement capacity and skills by developing permanent multidisciplinary centres to provide sustained inspiration, research, training and practical support for implementation and innovation. In the longer term, organisations need to build an infrastructure for quality improvement that includes an information system to track change and dedicated improvement leads across the organisation.

Health outcomes are improving rapidly in many countries. People are living longer and many previously fatal conditions have become treatable, enabling people to survive and retain a good quality of life. Although there have been many advances, influential reports and studies in many countries have drawn attention to serious safety and quality problems in healthcare. The World Health Organization has observed that such problems are systemic and permeate all healthcare systems, whether public or private.^1^

Healthcare frequently falls below expected standards and causes harm to patients. Studies of hospitals in many countries, carried out by reviewing medical records, have found that between 8 and 12% of patients admitted to hospital suffer harm that is sufficiently severe to require at least one additional day in hospital.^2^ There is much less information available about the safety and quality of mental health services,^3^ but there are some worrying findings. For instance, an Australian study found that adults with depression received an average of only 55% of recommended care^4^ and children with depression 33%.^5^

Patients will benefit from the development of new treatments for mental disorders and from increased resources for mental healthcare. However, health gains can also be achieved by improving the safety and reliability of current treatments. Improving the delivery of existing treatment may often bring much greater benefits than developing new treatments and technologies.^6^ To achieve this, clinical teams and organisations need to build capacity for sustained and systematic improvement.

## The nature of improvement

Quality improvement is often narrowly identified with a set of techniques adapted from industrial settings. They include the US Institute for Healthcare Improvement's Model for Improvement, which, among other things, combines measurement with tests of small change (plan–do-study–act cycles). These techniques provide a useful toolkit and are applicable to many problems, but more mature improvement organisations will draw on a much wider suite of methods when the occasion demands.

The most immediately effective quality and safety improvements have been those with a strong focus on a core clinical issue or a specific clinical process or pathway, often using checklists and ‘care bundles’ of interventions that increase the consistency of care.^7^ Many other types of intervention can be employed to help staff to work more effectively.^8^ For instance, medication errors have been reduced by standardising formularies and protocols, by including pharmacists in ward rounds and by introducing computerised prescribing. Errors can also be reduced by improving working conditions, for instance by minimising the interruptions and distractions that greatly increase propensity to error.^9^

## Centres of improvement

Quality improvement is often a small-scale, local activity in which each team painstakingly works out its own solution for each problem.^10^ The core quality improvement methods are aimed at the engagement of frontline staff, who are empowered to address problems in their own environment. This is certainly valuable but not sufficient to address complex, entrenched problems. Working in this isolated way means a lack of critical mass to support the right kinds of expertise, such as the technical skill in human factors or ergonomics necessary to engineer a process or devise a safety solution.^10^

Larger organisations can build improvement capacity and skills by developing permanent multidisciplinary centres to provide sustained inspiration, research, training and practical support for implementation and innovation.^11^ Small units exist within many hospitals and other healthcare organisations, but are often mainly concerned with regulation and compliance. The centres described in the series of papers on quality improvement in this issue of *BJPsych International* have a wider vision, however. They aim to systematically improve the safety and quality of care provided to patients and the working lives of their staff. While they are very different in size, scope and in their place in the healthcare system, they share some common characteristics. First, they are led by people who are passionate about improving healthcare. Second, they have support from senior managers and executives in their organisations or region. Third, they all provide education and training programmes in quality and safety improvement, which range from short introductory courses to deep programmes of study. Fourth, they are all open to wider learning both within and outside their organisations. Finally, they have all built trust within their teams and organisations to enable open discussion of safety and quality issues. In the longer term, organisations must also build an infrastructure for quality improvement that includes an information system to track change, dedicated improvement leads across the organisation, and an education programme and a department or an institute to support improvement.^12^

## Making a start

Many reading these papers will admire what has been achieved in Jönköping, Tuscany or other centres but think that this could not be achieved in their own environment. All these centres, however, began with a small group of enthusiastic people who tried to tackle immediate local problems. From such small beginnings, they eventually built the established centres described. Wherever you work and in whatever system, we hope these papers and these centres will inspire you and support you on your improvement journey.

## Supplementary material

For supplementary material accompanying this paper visit https://doi.org/10.1192/bji.2020.27. click here to view supplementary material
